# Female Mice Reaching Exceptionally High Old Age Have Preserved 20S Proteasome Activities

**DOI:** 10.3390/antiox10091397

**Published:** 2021-08-31

**Authors:** Irene Martínez de Toda, Suresh I. S. Rattan, Mónica De la Fuente, Lorena Arranz

**Affiliations:** 1 Department of Genetics, Physiology and Microbiology (Unit of Animal Physiology), Faculty of Biology, Complutense University, 28040 Madrid, Spain; imtcabeza@ucm.es (I.M.d.T.); mondelaf@bio.ucm.es (M.D.l.F.); 2 Institute of Investigation Hospital 12 Octubre, 28041 Madrid, Spain; 3 Department of Molecular Biology and Genetics, Århus University, 8000 Århus, Denmark; rattan@mbg.au.dk

**Keywords:** aging, healthy aging, exceptionally old, 20S proteasome, caspase-like activity, chymotrypsin-like activity

## Abstract

Oxidized, damaged and misfolded proteins accumulate during aging and contribute to impaired cell function and tissue homeodynamics. Damaged proteins are degraded by cellular clearance mechanisms like the 20S proteasome. Aging relates to low 20S proteasome function, whereas long-lived species show high levels. However, contradictory results exist depending on the tissue or cell type and it is unknown how the 20S proteasome functions in exceptionally old mice. The aim of this study was to investigate two proteasome activities (caspase-like and chymotrypsin-like) in several tissues (lung, heart, axillary lymph nodes, liver, kidney) and cells (peritoneal leukocytes) from adult (28 ± 4 weeks, *n* = 12), old (76 ± 4 weeks, *n* = 9) and exceptionally old (128 ± 4 weeks, *n* = 9) BALB/c female mice. The results show different age-related changes depending on the tissue and the activity considered, so there is no universal decline in proteasome function with age in female mice. Interestingly, exceptionally old mice displayed better maintained proteasome activities, suggesting that preserved 20S proteasome is associated with successful aging.

## 1. Introduction

Accumulation of damage in DNA, RNA and proteins is one of the universal features of aging. Whatever the causative agents and the source of the damage, increased molecular heterogeneity and the resulting impairment of functions at all levels of organization are hallmarks of aging [1,2,3]. In the case of proteins, damaged, modified and abnormal proteins accumulate, aggregate and interfere with normal cellular functions [4]. Therefore, an efficient cellular function requires balance between protein synthesis/folding, and degradation of misfolded or damaged proteins, termed proteostasis or proteodynamics [5].

The two main protein degradation systems are the autophagy pathway through the lysosomes and the proteasome system [5]. The proteasome is an evolutionary conserved multi-subunit protein complex [6]. The four-ringed 20S proteasome is the basic or core form, to which two 19S regulators are added to form the 26S proteasome [7]. Each eukaryotic 20S core particle has three pairs of proteolytic sites with distinct substrate specificities [8]. The β5 proteolytic sites are “chymotrypsin-like” (CT-like); the β2 sites are “trypsin-like” (T-like); and the β1 sites cleave after acidic residues (Glu, Asp) and are referred to as “post-acidic” PGPH (“post-glutamate peptide hydrolase”), or “caspase-like” (C-like). In the past, the 20S proteasome was seen as a mere component of the 26S proteasome complex, mediating degradation of ubiquitin-tagged proteins. New emerging evidence indicates that the 20S proteasome alone is responsible for cell clearance of abnormal, denatured or in general damaged proteins, and fine-tuned degradation of short-lived proteins [9]. In the absence of 19S regulators, the 20S proteasome specifically unfolds damaged or oxidized proteins in an ATP/ubiquitin-independent manner by recognition of hydrophobic domains exposed to the aqueous environment [7]. This seems to be the main function of the 20S proteasome [6,9] and therefore, the study of its activity is relevant in the context of aging.

Several studies indicate decline of the 20S proteasome activity with age that depends on at least three different mechanisms: decreased proteasome expression, alterations and/or replacement of proteasome subunits and formation of inhibitory cross-linked proteins [10]. Peptidase activities decrease with age in a variety of tissues and cells such as lymphocytes [11], fibroblasts [12] and muscle [13,14] from rodents, human lens [15] and fireflies [16]. However, sometimes contradictory results were reported, and some authors found no changes in any of the three peptidase activities with age [17] whereas others found decreased CT-like activity in liver from rats [17,18]. In human lymphocytes, decreased age-related specific activity of the 26 proteasome is associated with increased post-translational modifications, whereas levels and subunit composition are unaffected [19]. In turn, human aging epidermis show both decreased content and alterations in subunits contributing to decreased activity of the 20S proteasome [20,21].

Conversely, long-lived animals, such as long-lived bat species, show high expression levels of the 20S proteasome subunits. These are related to less accumulation of oxidized proteins and greater resistance to oxidative stress [22]. Similarly, naked mole rats (which are the longest living rodent species) display very high expression of the 20S proteasome, resulting in better oxidative stress resistance compared to other rodents [23]. Besides interspecies differences, the study of the 20S proteasome in exceptionally old individuals (which presumably experience “healthy aging”) in comparison to those that achieve average life span from the same species, can help further elucidate its role in aging and longevity. For example, fibroblast cultures from centenarians exhibit proteasome subunit expression, C-like activity and levels of oxidized proteins more similar to younger rather than older donors [24].

To gain further insight into the activity of the 20S proteasome in aging and longevity, the aim of the present work was to investigate two different proteasome activities (CT-like and C-like) in several tissues (heart, lung, liver, kidney, axillary lymph nodes) and cells (peritoneal leukocytes) from adult, old and exceptionally old BALB/c female mice.

## 2. Materials and Methods

### 2.1. Experimental Animals

We used 30 female BALB/c mice (Mus musculus), purchased from Harlan Ibérica (Barcelona, Spain), at a young adult age (28 ± 4 weeks). The mice were specifically pathogen free as tested by Harlan and according to the Federation of European Laboratory Science Associations recommendations. In our animal facility, placed at the Faculty of Biology (UCM), they were housed at 6 ± 1 per cage and maintained at a constant temperature (22 ± 2 °C) in sterile conditions inside an aseptic air negative pressure environmental cabinet (Flufrance, Cachan, France), on a 12/12 h reversed light/dark cycle (lights on at 8 pm). Mice had access to tap water and standard Sander Mus pellets (A04 diet; Panlab, Barcelona, Spain) ad libitum. Diet was in accordance with the recommendations of the American Institute of Nutrition for laboratory animals.

This cross-sectional study, defined as a study that takes place at a single point in time, was performed simultaneously on mice of different ages, namely adult (28 ± 4 weeks, *n* = 12), old (76 ± 4 weeks, *n* = 9) and exceptionally old (128 ± 4 weeks, *n* = 9), which had aged in our animal unit under the above specified conditions from the adult age. Each age group category was formed by animals that had been purchased in the same set. The exceptionally old mice had naturally achieved healthy and successful aging, since the average life span for females of BALB/c mice strain in our animal house was 99 ± 5 weeks [25]. The percentage of these females that reach exceptionally old age was approximately 7%–10%. Not all animals provided the full set of data. Mice were treated according to the guidelines of the European Community Council Directives (86/6091 EEC). They were sacrificed by rapid cervical dislocation during the dark phase of the cycle (08:00–10:00 a.m.). Heart, lung, liver, kidney, axillary lymph nodes and peritoneal leukocytes were removed aseptically and immediately frozen down at −80 °C until further processing.

### 2.2. Preparation of Tissue Lysates

To preserve proteasome activity, native protein lysates were obtained by resuspension of frozen tissue and cell pellets in buffer containing 50 mM NaCl, 10 mM HEPES pH 8, 500 mM Sucrose, 1 mM EDTA, 0.2% Triton 100X, 0.2 mM phenylmethanesulfonyl fluoride (PMSF) and 7.2 mM β-mercaptoetanol. Cell debris was removed by centrifugation and protein concentration was assessed in the supernatant using the bicinchoninic acid Protein Assay Kit protocol (BCA, Sigma).

### 2.3. Proteasome Activity Assay

Peptides used for determination of proteasome CT-like and C-like activities were Succinyl-leucine-leucine-valine-tyrosine-7-Amino-4-Methylcoumarin (Suc-LLVY-AMC) and Carboxybenzyl-Leu-Leu-Glu-7-Amino-4-Methylcoumarin (Z-LLE-AMC), respectively. Suc-LLVY-AMC or Z-LLE-AMC were added to a flat-bottom 96-well plate at a final concentration of 25 µM or 150 µM, respectively, together with the tissue/cell lysates adjusted to different protein concentrations, depending on the tissue, in order to measure linear kinetics of proteasome activity and ensure saturation was not reached during the course of the assay, in a final volume of 200 µL in 1 M HEPES pH 8. Protein amounts used were 60 µg for heart; 20 µg for liver, axillary nodes and peritoneal leukocytes; 10 µg for lung; and 2 µg for kidney. All measurements were made in triplicate, and samples for comparisons were run in the same test. For each individual and proteasome activity measurement, a negative control was performed in duplicate by adding a specific inhibitor of proteasome activity (MG132) at a final concentration of 400 µM. To detect the activity of the 20S proteasome, ATP was not added to the reaction mixtures.

The reading was performed in a plate reader with a 380 nm excitation filter and a 460 nm emission filter. The plate was incubated inside the fluorimeter for 50 min, and during this time, 25 reading cycles were performed, 1 cycle every 120 s. The respective activity in the presence of MG132 was subtracted from each sample to obtain the specific activity (fluorescence arbitrary units per time in seconds). Results were expressed as specific activity/mg protein.

Trypsin-like activity was not measured due to high interference of non-proteasome mediated trypsin-like activities in biological samples where the proteasome was not purified [26,27].

### 2.4. Statistical Analysis

Data are expressed as mean ± standard deviation (SD). The normality of the samples and the homogeneity of variances were checked by the Kolmogorov–Smirnov and Levene analyses, respectively. Differences due to age were studied through the one-way analysis of variance. The Tukey test with a level of significance set at *p* < 0.05 was used for post-hoc comparisons when variances were homogeneous, whereas its counterpart analysis Games-Howell set at the same significance level was used with unequal variances. Differences versus adult age are shown.

## 3. Results

**Figure 1:**
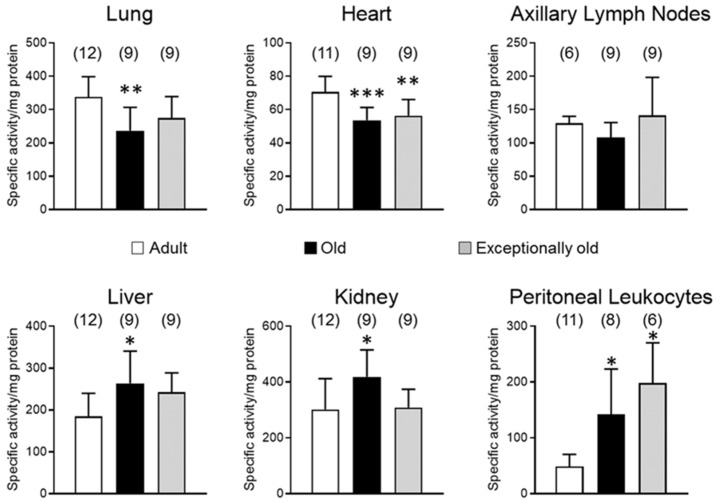
Age-related changes in caspase-like (C-like) activity in different tissues: Lung; heart; axillary lymph nodes; liver; kidney; peritoneal leukocytes. Adult (28 ± 4 weeks); old (76 ± 4 weeks); exceptionally old (128 ± 4 weeks). Numbers of animals used for each experimental group and biological sample are in brackets. * *p* < 0.05; ** *p* < 0.01; *** *p* < 0.001, with respect to adult mice.

Figure 1 shows results for C-like activity in adult, old and exceptionally old female mice. Old mice displayed lower C-like activity in lung (236.84 ± 69.87) and heart (53.60 ± 7.65) in comparison with adults (337.96 ± 60.45, 70.71 ± 9.22, respectively), whereas in liver, kidney and peritoneal leukocytes, old mice showed higher (263.74 ± 76.77, 418.85 ± 96.45, 141.87 ± 81.40, respectively) C-like activity than adults (184.77 ± 54.94, 301.85 ± 110.33, 48.79 ± 21.82, respectively; *p* < 0.05). Axillary lymph nodes showed unchanged values with age (129.44 ± 10.51 in adult, 108.17 ± 22.59 in old and 141.33 ± 57.12 in exceptionally old). Exceptionally old mice exhibited similar C-like activities to adults in lung (275.08 ± 63.90), liver (242.38 ± 46.56) and kidney (308.95 ± 65.25), plus axillary lymph nodes. Like old mice, exceptionally old mice showed lower (56.38 ± 9.66) C-like activity in heart (*p* < 0.01) and higher (198.33 ± 72.06) in peritoneal leukocytes (*p* < 0.05) than adults.

**Figure 2:**
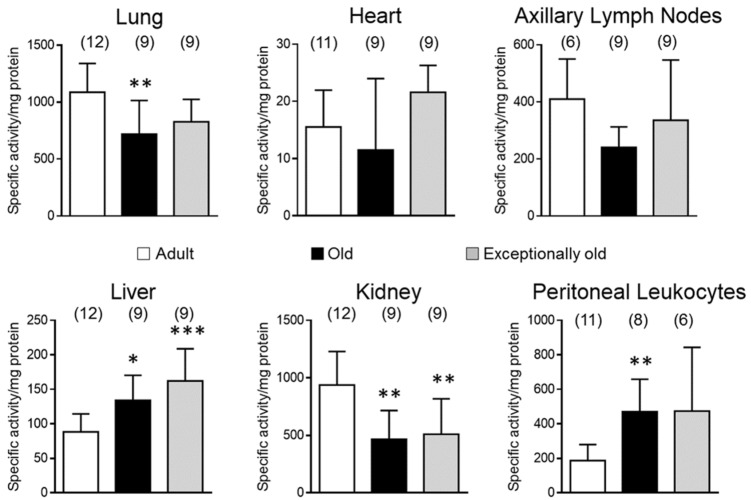
Age-related changes in chymotrypsin-like (CT-like) activity in different tissues: Lung; heart; axillary lymph nodes; liver; kidney; peritoneal leukocytes. Adult (28 ± 4 weeks); old (76 ± 4 weeks); exceptionally old (128 ± 4 weeks). Numbers of animals used for each experimental group and biological sample are in brackets. * *p* < 0.05; ** *p* < 0.01; *** *p* < 0.001, with respect to adult mice.

Figure 2 shows results for CT-like activity in adult, old and exceptionally old female mice. Old mice displayed lower CT-like activity in lung (719.04 ± 295.77) and kidney (466.85 ± 249.30) in comparison with adults (1086.47 ± 252.50, 938.35 ± 290.98, respectively; *p* < 0.01). Conversely, old mice had higher CT-like activity in liver (134.13 ± 36.24; *p* < 0.05) and peritoneal leukocytes (471.34 ± 187.64; *p* < 0.01) than adult mice (88.53 ± 26.05, 186.82 ± 93.60, respectively). No differences were found in heart and axillary lymph nodes with age (15.54 ± 6.43 in adult, 11.50 ± 12.48 in old and 21.59 ± 4.71 in exceptionally old in heart, and 409.61 ± 140.53 in adult, 240.17 ± 71.97 in old and 335.85 ± 211.08 in exceptionally old in axillary lymph nodes). Exceptionally old mice showed similar CT-like activity to adults in lung (826.51 ± 198.39) and peritoneal leukocytes (473.36 ± 370.80). Likewise, exceptionally old mice had unchanged CT-like activity in heart and axillary lymph nodes with respect to adults. CT-like activity showed similar trends to old in exceptionally old age in kidney (509.60 ± 307.95) and liver (162.26 ± 46.62) with lower (*p* < 0.01) and higher values (*p* < 0.001) respectively, compared to adult mice.

## 4. Discussion

The present study shows relevant and complex age-related changes in C-like and CT-like activities of the 20S proteasome. These changes show strong particularities depending on the organ and peptidase activity considered. Some proteasome activities were lower in old mice in comparison with adult mice, such as the C-like activity in lungs and heart, and the CT-like activity in lungs and kidney. These results are in agreement with previous studies [28,29] and with the general concept that age-related accumulation of damaged and dysfunctional proteins results, at least partially, from an impairment of the protein degradation pathways with age.

However, other proteasome activities were higher in old mice in comparison with adults, such as the C-like activity in liver, kidney and peritoneal leukocytes and the CT-like activity in liver and peritoneal leukocytes, in agreement with some previous studies [17,30]. The liver and kidney play key detoxifying functions, and peritoneal leukocytes are recruited into the peritoneal cavity to prevent peritoneal infection through activating pro-inflammatory cytokines and chemokines [31]. In these tissues, age-related high proteasome activities could reflect a compensatory mechanism to the high loads of oxidative stress and oxidized proteins, given that the proteasome activity is modulated by oxidative stress [32]. In fact, these tissues are also great reservoirs of antioxidant defenses that may help scavenging free radicals and contribute to lowering the levels of oxidized proteins [33,34]. Future work should evaluate these hypotheses and test to what extent the higher proteasome activities in liver, kidney and peritoneal leukocytes at old age are efficient in counteracting the accumulation of damaged proteins.

The net differential degree of age-related oxidative stress affecting tissues may be a relevant source of tissue-specific differences. Indeed, under normal conditions, the greater the proteasome activation, the greater the oxidative stress. However, if the oxidative stress is too high, the proteasome may be inhibited [35,36]. Additionally, the machinery of protein quality control is also tissue-specific and may contribute to variability in proteasome activity among tissues, including, for example, the expression of heat-shock proteins [37].

Of note, our observations do not ascertain the underlying mechanisms responsible for the age-related changes in 20S proteasome activities, e.g., proteasome subunit expression, translation and/or assembly, and/or regulation of activity by, for example, formation of inhibitory complexes [10]. Future work is required to clarify these aspects.

Overall, exceptionally old mice had similar peptidase activities to adult mice in most tissues investigated, suggesting that preserved 20S proteasome function correlates with achieving extreme old age. Exceptionally old mice showed lower C-like activity in the heart and CT-like activity in the kidney, and higher CT-like activity in the liver and peritoneal leukocytes, in similar trends to old mice. Lower activities in exceptionally old animals may suggest relatively disposable functions in the context of reaching successful aging, whereas higher/preserved activities could reflect adaptive mechanisms underlying healthy aging. Consistent with this idea, longevity was associated with higher CT-like activity in liver of naked mole rats compared to shorter-lived mice [38]. These interpretations should be considered with caution though, as the age-related changes in old and exceptionally old mice may occur at different rates and/or have different evolutions along the aging process. Future longitudinal studies will be needed to test those hypotheses. In addition, future work should investigate if the proteasome function in exceptionally old mice correlates inversely, in fact, with the level of accumulation of damaged proteins. Supporting this idea, a previous study demonstrated that peritoneal leukocytes from exceptionally old mice show higher basal levels of Hsp70 and lower accumulation of malondialdehyde, a marker of lipid peroxidative damage compared to old mice [39]. Further, protein oxidation markers in the brain and spleen from adult and exceptionally old mice showed similar levels, which were higher in old mice [25]. The mechanisms underlying better preserved 20S proteasome activity in exceptionally old mice should be the focus of future work.

As an additional limitation of our study, proteasome activities were only investigated in female mice. Female mice were prioritized over males to promote research on females, because of the ease of caging them together as mice are a social species, and because females of virtually all species including mammals live longer than males [40,41,42]. The longer lifespan in females has been associated to multiple factors, and for example the heterogametic sex has been suggested to be more likely to express undesirable morphological and physiological characteristics [40]. Accumulated knowledge demonstrates that estrogen upregulates expression of antioxidant enzymes via the estrogen receptor and MAPK activation, which results in the upregulation of expression of longevity-related genes [42]. In comparative studies using tissues like heart, old male rats showed increased protein oxidative damage compared to old females [43]. Future studies should expand gender comparisons along the aging process to additional tissues, including both protein oxidative markers and 20S proteasome activity, and investigate whether exceptionally old male mice display maintained proteasome activity as well.

In fact, a pulse of estrogen in ovariectomized mice aimed at mimicking late diestrus II/early proestrus, stimulated the turn-over of certain proteins, including estrogen receptor alpha and 20S proteasome chymotryptic activity in mammary glands in vivo [44]. In mammary glands from three-month-old female mice, the chymotryptic peptidase activity of the proteasome was elevated by about 40% during proestrus compared to the other stages [44]. It is uncertain whether the effects of estrogens during the estrus cycle extend to damaged proteins and additional tissues. To minimize potential effects of natural estrogen fluctuations in younger females, adult mice used here were seven months old, a stage where reproductive fertility starts to decay and diestrus stage is prolonged.

## 5. Conclusions

Together, our data show unique patterns of age-related changes in the 20S proteasome activity and no universal decline in old female mice. Exceptionally old female mice displayed well-preserved 20S proteasome activities. Although further analyses are needed to evaluate the role of the 20S proteasome during the aging process, our study provides evidence that exceptionally old mice have sustained activity which may help achieve successful aging.

## Data Availability

Data is contained within the paper.
